# Contribution of the Economic Crisis to the Risk Increase of Poor Mental Health in a Region of Spain

**DOI:** 10.3390/ijerph15112517

**Published:** 2018-11-09

**Authors:** Nayara Tamayo-Fonseca, Andreu Nolasco, Joaquín Moncho, Carmen Barona, María Ángeles Irles, Rosa Más, Manuel Girón, Manuel Gómez-Beneyto, Pamela Pereyra-Zamora

**Affiliations:** 1 Unidad mixta de investigación para el análisis de las desigualdades en salud y la mortalidad FISABIO-UA, University of Alicante, Campus de San Vicente del Raspeig s/n, Ap. 99, 03080 Alicante, Spain; nayara.tamayo@ua.es (N.T.-F.); joaquin.moncho@ua.es (J.M.); giron@icali.es (M.G.); pamela.pereyra@ua.es (P.P.-Z.); 2 Conselleria de Sanitat Universal i Salut Pública, Foundation for the Promotion of Health and Biomedical Research in the Valencian Region (FISABIO), Generalitat Valenciana, 46020 Valencia, Spain; barona_car@gva.es (C.B.); irles_ang@gva.es (M.Á.I.); mas_ros@gva.es (R.M.); 3 CIBERSAM, Instituto de Salud Carlos III, 28029 Madrid, Spain; manuel.gomez-beneyto@uv.es; 4 Teaching Unit of Psychiatry and Psychological Medicine, Department of Medicine, University of Valencia, 46010 Valencia, Spain

**Keywords:** economic recession, mental health, health status disparities, Spain, GHQ

## Abstract

Previous research suggests that the economic crisis can affect mental health. The purpose of this study was to analyse the association of risk of poor mental health with various socioeconomic, demographic, health, quality of life, and social support variables; and to evaluate the contribution of socioeconomic variables most affected by the beginning of the economic crisis (employment situation and income) on the changes in the prevalence of the risk of poor mental health between 2005 and 2010. A study of prevalence evolution in adult population residents of the Valencian Community in the Spanish Mediterranean was conducted. We studied 5781 subjects in 2005 and 3479 in 2010. Logistic regression models have been adjusted to analyse the association between variables. A standardisation procedure was carried out to evaluate which part of the changes in overall prevalence could be attributed to variations in the population structure by age, sex, employment status, and income between the years under study. The prevalence of GHQ+ increased from 2005 to 2010, in both men and women. Several variables were closely associated with the risk of poor mental health (sex, age, country of birth, number of nonmental chronic diseases, social support, disability, cohabitation in couple, employment status, and income). The changes produced as a result of the onset of the economic crisis in income and unemployment (increase in low income and in unemployment rates) contributed to the increase of poor mental health risk. This could confirm the sensitivity of mental health to the economic deterioration caused by the crisis.

## 1. Introduction

Mental health problems affect at least one in four people worldwide at some time in their lives [1]. Neuropsychiatric disorders are the second greatest cause of the burden of disease in Europe and are the greatest cause of years lived with disability [1].

Since the beginning of the economic recession in Europe, various studies have been pointing out the relationship between crisis and poor mental health [2], as well as the various determinants that may be affecting it [3,4,5,6,7]. The majority of studies on the subject have focused on the analysis of psychological and behavioural morbidity, with predominance of countercyclical studies on depression, anxiety, or violent behaviour and their relation with job loss as a risk factor. Additionally, other studies have analysed changes in some behavioural risk factors, suggesting that in a situation of prolonged economic depression, it is likely for higher rates of alcoholism and smoking or substance abuse to be observed [7,8,9]. On the other hand, some authors have also described the impact of the economic crisis on general mortality, as well as on some specific causes, such as suicides or external causes, suggesting countercyclical as well as procyclical effects [7,10,11,12,13].

In Spain, following this trend, a number of studies have been published in recent years describing the association and impact of the economic crisis and poor mental health [14,15,16,17,18]. In general, it has been identified that economic changes have intensified the social exclusion and marginalisation of people with mental health problems, especially in men and, to a greater extent, in those with low levels of education [11,14,19], in people affected by mortgage-related financial difficulties or evictions [9,17], as well as among immigrants [20,21,22]. Unemployment has also been shown to have a significant negative impact on overall health and mental health and has been described as the main risk factor for mental disorders [16,17]. Despite the recent literature on this subject, several limitations have been described in the studies, as well as the need to generate new knowledge from the basic sciences, and the epidemiological method to establish the association between economic decline and the effect on mental, behavioural, or somatic health has also been highlighted [7,18,23].

Finally, the impact of the financial crisis on Spain, unlike other countries (such as Ireland or Greece) was delayed until the end of 2011. This was due partly to the cushioning of the highly developed popular social support network and partly to the governmental contentious strategy that, despite the increase of the debt, allowed to maintain the benefits of the welfare state and national social protection [11]. Nevertheless, Spain has also been described as one of the countries of the European Union enduring the worst consequences due to the weakening of its economic activity and the deterioration of its public finances [9]. The Valencian Community (thereafter VC) is one of the 17 regions in which Spain is structured, located in the Mediterranean coast. It has been one of the regions most affected by the crisis, along with Andalusia, Castilla-La Mancha, and Murcia, unlike other, less affected territories such as Navarre, La Rioja, and the Basque Country. While poverty has grown in Spain to 8% between 2008 and 2011, in the VC it has increased up to 18%, ranking second, only behind the Canary Islands [24].

On the other hand, although the prevalence of mental disorders in Mediterranean countries has been described as inferior to the countries of the north of Europe [25], it is necessary to find out the impact on the health due to the much more pronounced economic changes in these countries.

Given the context of changes in Spain in recent years—related to the economic crisis—and the lack of evidence from studies that have suggested a specific methodology to evaluate the contribution of socioeconomic changes introduced since the beginning of the economic crisis, the objectives of this study are to analyse the association of the risk of poor mental health with various demographic, socioeconomic, health status, quality of life, and social support variables; and, specifically, to evaluate the contribution of socioeconomic variables most affected by the economic situation (employment and income) on changes in the prevalence of poor mental health risk between 2005 and 2010 (period including the beginning of the economic crisis in Spain) in the general adult population of the VC, a Mediterranean region of the Spain.

## 2. Materials and Methods

### 2.1. Design, Population, and Sample

This is a study of prevalence evolution of poor mental health risk and associated factors in adult population, over 15 years of age, between 2005 and 2010.

The total sample sizes were 5781 subjects in 2005 and 3479 in 2010, living in the VC, an autonomous community with just over five million inhabitants in 2008. The samples corresponded to the adults of the Health Surveys of the Valencian Community (thereafter ESCV) carried out every year under study, being representative of the non-institutionalised general adult population of the VC. The subjects of the samples were selected using a complex sampling design that assigned each subject a weighting according to their representativeness. The weights were included in the ESCV databases provided by the Health Plan Service of the Conselleria de Sanitat of the Generalitat Valenciana (the Health Ministry of the Valencian Government). Details about the survey methodology (sample design, sample size, sampling procedure, consent, ethics, etc.) have been published elsewhere [26,27].

This research uses two transversal observational studies based on administrative data. An informed consent was required from every participant in the Health Surveys of the Valencian Community by the Valencian Health Authorities. According to national regulations, data from National or Regional Health Surveys [28] are public in Spain and the Valencian Health Authorities are responsible for and guarantee confidentiality and anonymity, making the approval of an ethics committee unnecessary. The researchers only had access to public data that had been rendered anonymous, and so this research poses no ethical issues.

### 2.2. Variables

The variable answer was ‘Case at risk of poor mental health’ with yes or no as possible results. In order to construct this variable, the questions corresponding to the 12-item General Health Questionnaire (GHQ-12) were used, assigning to each of the items that compose the score 0 if the answer was 0 or 1, and 1 if the answer was 2 or 3. The subject at risk of poor health (GHQ+) was classified if the sum of the scores of the 12 items was equal to or greater than 3.

In addition, demographic explanatory variables were included for both years: Sex (woman, man), age (16–44, 45–64, 65–84, ≥85 years); variables of socioeconomic level: Country of birth (Spain, abroad), level of education (university, professional training/secondary school, primary, without studies), employment status (employed, unemployed, other situations—student, housekeeping, retired, other), personal self-perceived income level (medium–high, low), occupational class (manual, nonmanual); Health status variables: Presence of a nonmental chronic disease (yes, no), number of nonmental chronic diseases, presence of a disability (yes, no), score of self-perceived quality of life questionnaire or EuroQoL-5D [29]; and variables related to social support: Marital status (single, married, separated/divorced, widowed), cohabitation with a partner (yes, no). Both the response variable and all the explanatory variables were measured equally in both the 2005 and 2010 surveys.

### 2.3. Methods of Analysis

Number, percentage, and 95% confidence interval (95%CI) of subjects in each category of the explanatory variables were calculated for the qualitative variables and for each year of the survey. Prevalence and 95%CI of the GHQ+ result altogether and in each category of the explanatory variables were calculated by analysing with the Chi-Square test the significance of the association between GHQ+ and each variable, for each sex, separately.

For the quantitative variables and for each year, the number, average value, and 95%CI in each category of the response were calculated, analysing with the t-test the significance of the differences of averages, separating by sex.

For the analysis of association of GHQ+ with the explanatory variables, logistic regression models for both sexes were adjusted together. As a measure of association, the Odds Ratios (OR) were calculated between the GHQ score and the explanatory variables, as well as their corresponding 95%CI, first in simple analysis and then adjusting for all variables. The statistical significance of the interaction of all variables with sex was checked to verify the homogeneity of the models in both sexes. All the analyses have taken into account the complex sampling design, using the weighting of the subjects of each sample. Statistical analysis was performed using the SPSS v.18 program. The level of significance was 0.05 in all analyses.

In order to study the extent to which changes in the employment situation and income could have affected the observed changes in the prevalence of risk of poor mental health from 2005 to 2010, the percentage distribution of the sample by these variables in 2005 was projected on each one of the levels and sublevels of the categories of variables in 2010, adjusting for age, sex, and country of birth. The variables included in this analysis were sex (male, female), age (in 3 categories, 16 to 44, 45 to 64, and 65 or over), country of birth (Spain, other), income (in 2 categories; high–medium, low) and employment status (in 3 categories; work, unemployed, other situations), giving rise to a total of 72 strata or different levels (2 × 3 × 2 × 2 × 3 = 72).

The projection was made as follows:

- Calculation of the frequencies that would have been observed in the 2010 sample in every stratum, in case the percentage distribution observed in 2005 would have not changed:
  $$P_{e_{i}}^{2010}=\frac{P_{i}^{2005}}{\sum P_{i}^{2005}}\left(\sum P_{i}^{2010}\right)\;;\;i=1,2,3\ldots ,72,$$
  where:
  $P_{i}^{2005}$ = Frequency in stratum *i* in 2005;
  $P_{i}^{2010}$ = Frequency in stratum *i* in 2010;
  $P_{e_{i}}^{2010}$ = Frequency that should have been observed in stratum *i* in 2010 if percentage distribution would have changed as regards 2005.
- Calculation of the observed GHQ+ prevalence in 2010, in every disaggregation stratum:
  $$prev_{i}^{2010}=\frac{{n_{GHQ+}}_{i}^{2010}}{P_{i}^{2010}};\;i=1,2,3\ldots ,72,$$
  where:
  ${n_{GHQ+}}_{i}^{2010}$ = Number of GHQ+ cases observed in the stratum *i* in 2010;
  $prev_{i}^{2010}$= Prevalence of GHQ+ in the stratum *i* in 2010.
- Calculation of expected cases of mental health and its prevalence in every stratum:
  $${n_{e\_GHQ+}}_{i}^{2010}=P_{e_{i}}^{2010}\cdot prev_{i}^{2010}=P_{e_{i}}^{2010}\cdot \frac{{n_{GHQ+}}_{i}^{2010}}{P_{i}^{2010}};\;i=1,2,3,\ldots ,72,$$
  $$prev_{e,i}^{2010}=\frac{{n_{e\_GHQ+}}_{i}^{2010}}{P_{e_{i}}^{2010}},$$
  where:
  ${n_{e\_GHQ+}}_{i}^{2010}$ = Number of expected cases of GHQ+ in the stratum *i* in 2010 if percentage distribution would not have changed as regards 2005;
  $prev_{e,i}^{2010}$ = Expected prevalence in the stratum *i* in 2010 if percentage distribution would not have changed as regards 2005.
- Finally, the overall expected prevalence in 2010 was calculated if the percentage distribution with respect to 2005, disaggregated by sex, had not been changed, adding all previous cells, as follows:
  $$prev_{e,total}^{2010}=\frac{\sum {n_{e\_GHQ+}}_{i}^{2010}}{\sum P_{e_{i}}^{2010}}.$$

When comparing observed versus expected prevalence, in 2010, this standardisation procedure would make it possible to distinguish between: (1) The change in overall prevalence due to variations in population structure from 2005 to 2010 in terms of variables considered, and (2) the change due to the different period considered that would not be justified by the variations in the population structure.

## 3. Results

### 3.1. Risk of Poor Mental Health and Associated Variables

For 2005, 5781 subjects were analysed, 2855 of which (49.5%) were men and 2926 (50.5%) were women. The prevalence of risk of poor mental health was 20.0%, being 16.2% in men and 24.0% in women.

In 2010, we analysed 3479 subjects, 1702 of which (48.9%) were men and 1777 (51.1%) women. The prevalence of risk of poor mental health increased to 27.8%, being 25.4% in men and 30.6% in women.

In 2005, the highest prevalence of GHQ+ was found among older men, without studies, no employment status, low income level, presence of some chronic nonmental illness, presence of some disabilities, marital status separated, divorced, or widowed, and those with poor social support. In 2010, the most frequent profile was those born outside Spain, unemployed labour status, low income level, manual workers, presence of some chronic nonmental illness, presence of a disability, separated civil status, divorced or widowed, single, and those with poor social support (Table 1).

Regarding women (Table 2), the profile of those with the highest prevalence of poor mental health risk in 2005 was of an older person, without studies, unemployed, low income, presence of chronic nonmental illness, presence of a disability, widowed, single, and those with poor social support. In 2010, they were the most elderly, without studies, unemployed, low income level, presence of some chronic mental illness, presence of chronic nonmental illness, presence of a disability, widowed, and those with poor social support.

For both years, in both men and women, quantitative explanatory variables present significantly worse average values in subjects at risk of poor mental health (Table 3).

Overall, for both men and women, prevalence of poor mental health risk increased from 2005 to 2010 in most of the categories of variables studied, in line with the overall increase in prevalence.

Table 4 shows the ORs and 95%CI risk association of the poor mental health with the other variables (only those that presented significant association in one year), for both sexes together. In the two years, a similar logistic multivariate model is reached. It can be observed that the association of the labour situation and the level of income remain significant after adjusting for the remaining variables in both years, with high ORs for the categories of ‘unemployed’ and ‘low income’. The country of birth was not significant in 2005 after adjusting for the rest, but was so in 2010.

### 3.2. Impact of the Crisis and Changes in Prevalence

Table 5 shows the results corresponding to the change in the prevalence of poor mental health between the years 2005 and 2010. It is observed that if the population structure in terms of age, country of birth, income level, and employment status had not changed, the total prevalence of poor mental health expected in 2010 would be 21.4% (95CI% 19.5–23.3) in men and 28.5% (95CI% 26.4–30.6) in women, compared to the 25.4% in men and 30.6% in women actually observed. Consequently, the difference between these expected prevalence values and those actually observed in 2010 would be related to changes in population structure. Given that the prevalence of the risk of poor mental health observed in 2010 was 25.4% in men and 30.5% in women, the difference was higher than expected and, therefore, the observed prevalence excess attributable to changes in population structure would be around 4.0% in men and 2.1% in women in absolute terms. These values would translate, in relative terms, into a contribution of 43.5% and 31.8% of the increase in prevalence as attributable to changes in population structure, while the rest of the increases would be attributable to changes in other explanatory factors of the risk of poor mental health.

In order to explain the changes in population structure, Table 5 shows its distribution in each of the categories of variables considered. It can be seen that the distribution in 2005 and 2010 is similar by age group, in both men and women. In the case of the country of birth, there is an increase in absolute terms of 4.2% of the foreign population in men (from 10.1% to 14.3%) and around 2.0% in the case of women (from 10.4% to 12.4%). However, the greatest differences are detected in variables such as employment status and income. In the case of income, there is an increase in the population with less income of 11.1% in men and 9.3% in women in absolute terms, whereas for the labour situation, there is an increase in the unemployed population of 11.9% in men and 6.3% in women. It would be worth noting that the prevalence of poor mental health associated with these levels is the highest for the corresponding variable. In men, it can be observed that in the low-income category, the observed prevalence is 23.5% in 2005 and 33.5% in 2010 (the adjusted one in 2010 would be 30.0%), while regarding those in unemployment it is 34.4% in 2005 and 37.3% in 2010 (the adjusted one in 2010 would be 34.3%). Something similar occurs in the case of women, where the prevalence of poor mental health in the low-income categories is 33.6% in 2005 and 38.5% in 2010 and in unemployed women of 27.5% and 44.1%, respectively.

## 4. Discussion

This study has shown, firstly, that various variables regarding the demographic and socioeconomic context, as well as the areas of social support and health and quality of life were closely associated with the risk of poor mental health. This is particularly so regarding: Sex (worse in women), age (worse among the most elderly), country of birth (worse in foreigners), number of chronic nonmental illnesses (worse to a greater number), disability (worse if there is a presence of disability), quality of life (worse to worse score), social support (worse to worse score), cohabitation in couple (worse if not), employment situation (worse in unemployment), and income (worse if low income). These results coincide with some studies in Spain [5,6,30] that found the strongest predictors associated with poor mental health to be: Being a woman, having chronic illness, and having poor perception of health and quality of life and limited activity. However, the age in some studies had an inverse sense (worse in younger) [5,30]. 

Regarding changes in the prevalence in the two periods (before and during the crisis), this situation of increased risk has already been described in other studies that evaluate the impact on mental health in periods of economic recession in various countries of the world [18] as well as in Europe [31,32] or in Spain [9,10,15]. Our data reflect much higher prevalence than that found in England [32], and the latest epidemiological studies in Spain hardly show any changes between periods [14,16]. They display only a small increase in the prevalence of ill-health in men, passing from 14.7% in 2006 to 16.9% in 2011–2012 and, in the opposite direction, a reduction in women from 24.6% in 2006 to 22.7% in 2011–2012 is also described [14]. These differences could be explained in part because of the sensitivity of the instrument to collect the disorders, age periods, and intervals analysed and variation of diagnostic categories, or also due to selection or recall bias. They could also be explained by the impact of the crisis on pre-existing regional inequalities, the dampening of the social apparatus, the differences in unemployment rates since the first years of the crisis, the public indebtedness and the type of economy in the region, etc. It should be noted that the VC was already among the regions with the highest prevalence of risk of poor mental health in Spain. This can be partly explained by characteristics such as socioeconomic level, level of education, unemployment and immigration rates, and development of mental health care resources [33].

Regarding sex, women generally had worse results; however, when taking into account the changes in the period, there is a greater increase in the prevalence in men. In the literature, it has been described that in periods of crisis or recession, men show a greater increase in risk compared to women [7,32,34] and partly explained due to the impact of unemployment as a risk factor of mental health worsening [14]. These differences may be attributed to the relation between work and social role of the man as main supporter with high family burdens [35]. Despite this, it is necessary to highlight the starting high prevalence in women, possibly explained by factors such as the status of women in society, their workload, lower economic resources, lack of autonomy, lack of social support, and in some cases, the violence, overburden, and stress that they experience and that contribute to their poor health [36]. Studies in Spain have shown worse mental health in older women, immigrants from a low-income country [33], from rural areas [37], with increase in family burden [38], with obesity [39], and who have experienced different types of intimate partner violence [40]. In our study, we have found among unemployed women the highest prevalence of poor mental health and the greatest increases over the expected prevalence.

It is necessary to take into account this aspect, since in Spain a greater impact of the crisis has been described in women with higher rates of unemployment, part-time employment, and precarious and lower incomes, in comparison with men’s similar working hours [41].

When analyzing the country of birth, our results coincide with what is described in other studies. These describe an increased risk of poor mental health among immigrants in the wake of the crisis [20,21,22]. This is partly due to the fact that they are a group with high social and economic vulnerability, as evidenced by the very high rates of unemployment among the foreign population in Spain and in the VC. Unemployment reach quotas of up to 41% among immigrants from non-EU countries [42]. To this, the persistence of occupational segregation should be added, as well as economic and labour instability, family burdens, discrimination, lack of social support, and precarious income, among others.

Second, this study has demonstrated the importance of the contribution of the socioeconomic variables most affected by the onset of the economic crisis (employment situation and income) on changes in the prevalence of the risk of poor mental health between 2005 and 2010 in the population of the VC. Thus, the employment situation and the rent presented substantial changes from 2005 to 2010, increasing the number of unemployed and low income.

This study presents unpublished results rarely evaluated in the current studies on this subject. Unlike other studies that have used “the pooled data” of the two health surveys analysed in their models to evaluate changes in mental health prevalence in two periods (before and during the crisis) in Spain [14,16], we wanted to analyse the contribution of the effect of changes in the population structure on the excess prevalence of GHQ+. The analysis has taken into account the variables that have modified their distribution among the population in the second survey and coinciding with the changes in the economic and social situation of the VC (for example with the rise in unemployment and low income). In this regard, this analysis has made it possible to quantify the contribution, adjusted by age and sex, of these variables to the increases in the prevalence of risk of poor mental health from 2005 to 2010 in men and women in 43.5% and 31.8%, respectively, of the total increase that occurred in the prevalence. However, the rest should be explained by other factors. This could confirm the sensitivity of mental health to the economic deterioration caused by the crisis.

According to a WHO report, the crisis can deteriorate health through reductions in household financial security, especially as a result of job losses [8]. It has been described that unemployment is the determinant with the most stressing effect in life [43], which carries multiple health risks [7,12,19,34], and as the main factor in the appearance of problems such as anxiety, insomnia, depression, and dissociative and self-injurious behaviours that can cause the first mental health problems in a healthy person [34]. In addition, several studies have shown an increase of a 2–7-fold risk of suffering problems or symptoms associated with depression and anxiety about loss of work [7,19], as well as its detrimental effect over time [44]. One of the main consequences of the economic crisis in Spain has been the increase in unemployment. To date, Spain occupies the first position in relation to the unemployment rate vis-à-vis all EU countries since 2008 [45]. In the VC, the evolution of unemployment has shown a trend similar to the general Spanish one, although with rates higher than the national average [42,46], which shows a continued and chronic trend of unemployment both in Spain and in the VC.

Although our data are in line with the results of other studies in Spain, which have confirmed that unemployed people have higher levels of depression than the employed [47] and that the increase in unemployment is an important risk factor that could be related to the increase in demand for primary care [9,15], our findings do not coincide with those found in England, which found that changes in the mental health of the population do not seem to be entirely mediated by changes in the unemployment rate or household income [32]. One possible explanation could be that in the VC, there were much more drastic changes in unemployment rates (from 8.6% in 2006 up to 25% in 2012 in Spain vis-à-vis unemployment rates of 3% in England), a reduction in social and health services as part of the austerity policy to reduce debt, and a delay in government intervention strategies that made the impact more pronounced than in England.

### Strengths and Limitations

One of the strengths of this study has been its sample size and the representativeness of the sample with respect to the general population. Furthermore, the study refers to the general population of over 15 years, covering a wide range of age. Another strength of the study is the use of data from the 2005 and 2010 ESCV survey, designed and validated to obtain population information on the variables studied and with little lack of response. This study does not include an institutionalised population, so there may be an underestimation of mental disorders, since a high prevalence of mental health problems in nursing homes and residences has been described [30]. On the other hand, cross-sectional studies cannot identify the direction of associations, for example, between mental health and work status or other variables. Regarding the instrument, it should be emphasised that the General Health Questionnaire is not suitable for assessing chronic disorders, but it does allow certain “mental health problems” [48] to be identified. Other studies also point out that since GHQ is a screening instrument and not a diagnostic tool, and more sensitive than specific, it may overestimate the existence of mental health problems [33]. Other variables described in the literature and related to poor mental health have not been taken into account in the analyses, which may also be explaining a greater risk due in part to the fact that the intention of the choice of variables was directed to those related with the socioeconomic scope of the current crisis.

## 5. Conclusions

The prevalence of poor mental health risk increased substantially in the VC from 2005 to 2010. Several variables were closely associated with this: Sex, age, country of birth, chronic diseases, disability, quality of life and social support, and employment status and income. Nevertheless, there was no interaction of any variable with sex. On the other hand, employment situation and rent presented substantial changes from 2005 to 2010, increasing the number of unemployed and the low income. Thus, the contribution, adjusted for age and sex, of these variables to increases in the prevalence of risk of poor mental health, from 2005 to 2010 can be quantified in men and women by 43.5% and 31.8%, respectively, of the total increase that occurred in the prevalence. The rest should be explained by other factors. As a consequence, this could confirm the sensitivity of mental health to the economic deteriorations caused by crises.

## Figures and Tables

**Table 1:** Frequencies (*n*), percentages (%) of population distribution, and observed prevalence (P_o_) × 100 and 95% confidence interval (95%CI) of the risk of poor mental health, according to categories of explanatory variables and according to the year of the survey. Men.

MEN		2005						2010					
		*n*	%	95%CI	P_o_	95%CI	*p* *	*n*	%	95%CI	Prev	95%CI	*p* *
Total		2846	100	------	16.2	(14.8–17.6)	------	1702	100	------	25.4	(23.3–27.5)	------
Age	16–44	1588	55.8	(53.4–58.2)	13.5	(8.9–18.1)	<0.001	919	54.0	(50.8–57.2)	25.4	(19.8–30.9)	0.104
	45–64	787	27.7	(24.5–30.8)	16.8	(10.4–23.1)		497	29.2	(25.2–33.2)	25.2	(17.5–32.8)	
	65–84	438	15.4	(12.0–18.8)	21.9	(13.6–30.2)		257	15.1	(10.7–19.5)	23.7	(13.1–34.4)	
	≥85	33	1.2	(0.0–4.8)	54.5	(31.5–77.5)		29	1.7	(0.0–6.4)	44.8	(17.8–71.9)	
Country of birth	Spain	2420	89.9	(88.7–91.1)	16.4	(12.7–20.0)	0.562	1460	85.7	(83.9–87.5)	24.2	(19.7–28.6)	0.007
	Abroad	273	10.1	(6.6–13.7)	16.1	(5.3–27.0)		243	14.3	(9.9–18.7)	32.5	(22.2–42.8)	
Chronic disease	No	1739	61.1	(58.8–63.4)	10.0	(5.5–14.5)	<0.001	1039	61.0	(58.1–64.0)	20.9	(15.5–26.3)	<0.001
	Yes	1107	38.9	(36.0–41.8)	25.8	(20.8–30.9)		663	39.0	(35.2–42.7)	32.4	(26.2–38.7)	
Disability	No	2459	86.4	(85.1–87.8)	12.5	(8.8–16.2)	<0.001	1369	80.4	(78.3–82.5)	10.4	(5.4–15.4)	<0.001
	Yes	386	13.6	(10.2–17.0)	39.1	(31.3–46.9)		334	19.6	(15.4–23.9)	86.2	(82.2–90.2)	
Level of studies	University	413	14.7	(11.3–18.1)	10.9	(1.8–20.0)	<0.001	316	18.6	(14.3–22.9)	20.9	(11.1–30.7)	0.064
	Prof. train/high school	675	24.0	(20.8–27.3)	12.6	(5.5–19.6)		745	43.8	(40.2–47.4)	24.6	(18.3–30.8)	
	Elementary school	1698	60.4	(58.1–62.8)	18.4	(14.1–22.7)		493	29.0	(25.0–33.0)	27.8	(20.3–35.3)	
	No qualifications	23	0.8	(0.0–4.5)	47.8	(18.3–77.3)		147	8.6	(4.1–13.2)	30.6	(17.1–44.1)	
Employment	Employed	1866	66.1	(64.0–68.3)	11.5	(10.1–12.9)	<0.001	878	51.6	(48.3–54.9)	20.2	(17.5–22.9)	<0.001
situation	Unemployed	128	4.5	(0.9–8.1)	34.4	(26.2–42.6)		279	16.4	(12.1–20.7)	37.3	(31.6–43.0)	
	Other	827	29.3	(26.2–32.4)	23.8	(20.9–26.7)		544	32.0	(28.1–35.9)	27.6	(23.8–31.4)	
Income level	Medium–high	1757	68.7	(66.5–70.9)	12.6	(11.0–14.2)	<0.001	861	56.6	(53.3–59.9)	16.4	(13.9–18.9)	<0.001
	Low	800	31.3	(28.1–34.5)	23.5	(20.6–26.4)		660	43.4	(39.6–47.2)	33.5	(29.9–37.1)	
Occupation	Nonmanual work	1184	62.1	(59.3–64.8)	13.8	(8.5–19.1)	0.076	518	36.8	(32.7–41.0)	18.5	(10.8–26.3)	<0.001
	Manual work	724	37.9	(34.4–41.5)	10.9	(4.0–17.8)		889	63.2	(60.0–66.4)	29.1	(23.6–34.7)	
Marital status	Single	989	34.8	(31.9–37.8)	14.9	(9.1–20.6)	<0.001	557	32.7	(28.8–36.6)	24.6	(17.4–31.8)	<0.001
	Married	1668	58.7	(56.4–61.1)	15.6	(11.2–20.1)		1045	61.4	(58.4–64.4)	23.6	(18.3–28.9)	
	divorced/separated	116	4.1	(0.5–7.7)	25.0	(9.2–40.8)		56	3.3	(0.0–8.0)	51.8	(33.6–70.0)	
	Widower	67	2.4	(0.0–6.0)	32.8	(13.2–52.5)		44	2.6	(0.0–7.3)	43.2	(20.9–65.5)	
Living with a	Yes	1750	61.7	(59.4–64.0)	15.5	(11.2–19.8)	0.464	961	56.5	(53.3–59.6)	22.5	(16.9–28.0)	0.002
partner	No	1087	38.3	(35.4–41.2)	17.2	(11.8–22.6)		741	43.5	(40.0–47.1)	29.1	(23.1–35.2)	
Social support	Good support	2636	93.5	(92.5–94.4)	14.0	(10.5–17.6)	<0.001	1639	96.2	(95.3–97.2)	24.1	(19.9–28.3)	<0.001
	Bad support	184	6.5	(3.0–10.1)	46.2	(35.6–56.8)		64	3.8	(0.0–8.4)	57.8	(41.9–73.7)	

(*) *p*-values of the Chi-square test to check the significance of the differences among categories.

**Table 2:** Frequencies (*n*), percentages (%) of population distribution, and observed prevalence (P_o_) × 100 and 95% confidence interval (95%CI) of the risk of poor mental health, according to categories of explanatory variables and according to the year of the survey. Women.

WOMEN		2005						2010					
		*n*	%	95%CI	P_o_	95%CI	*p* *	*n*	%	95%CI	P_o_	95%CI	*p* *
Total		2905	100	------	24.0	(22.4–25.6)	------	1777	100	------	30.6	(28.5–32.7)	------
Age	16–44	1479	50.9	(48.4–53.5)	19.7	(15.2–24.3)	<0.001	867	48.8	(45.5–52.1)	28.0	(22.4–33.7)	0.001
	45–64	814	28.0	(24.9–31.1)	26.7	(20.8–32.5)		524	29.5	(25.6–33.4)	31.1	(24.0–38.2)	
	65–84	542	18.7	(15.4–21.9)	29.7	(22.6–36.8)		326	18.3	(14.1–22.5)	32.5	(23.6–41.4)	
	≥85	70	2.4	(0.0–6.0)	32.9	(13.7–52.1)		60	3.4	(–1.2 to 7.9)	51.7	(34.1–69.3)	
Country	Spain	2452	89.6	(8.4–90.8)	23.9	(20.5–27.4)	<0.001	1557	87.6	(86.0–89.3)	29.1	(24.9–33.3)	0.001
of birth	Abroad	284	10.4	(6.8–13.9)	21.5	(11.2–31.8)		220	12.4	(8.0–16.7)	40.9	(30.8–51.1)	
Chronic	No	1412	48.6	(46.0–51.2)	14.2	(9.4–19.1)	<0.001	980	55.1	(52.0–58.3)	25.2	(19.8–30.6)	<0.001
Disease	yes	1493	51.4	(48.9–53.9)	33.0	(28.8–37.1)		797	44.9	(41.4–48.3)	37.1	(31.6–42.6)	
Disability	No	2450	84.3	(82.9–85.8)	19.8	(16.3–23.4)	<0.001	1271	71.8	(69.4–74.3)	23.8	(19.0–28.6)	<0.001
	Yes	455	15.7	(12.3–19.0)	45.7	(38.9–52.5)		498	28.2	(24.2–32.1)	48.2	(41.9–54.5)	
Level of studies	University	451	15.7	(12.4–19.1)	17.5	(9.1–25.9)	<0.001	310	17.5	(13.2–21.7)	24.8	(15.2–34.5)	<0.001
	Prof. train/high school	598	20.8	(17.6–24.1)	20.7	(13.6–27.9)		738	41.6	(38.0–45.1)	26.4	(20.2–32.6)	
	Elementary school	1750	61.0	(58.7–63.3)	26.1	(22.0–30.1)		505	28.4	(24.5–32.4)	37.0	(30.1–44.0)	
	No qualifications	70	2.4	(0.0–6.1)	37.1	(18.6–55.7)		223	12.6	(8.2–16.9)	37.2	(26.8–47.6)	
Employment	Employed	1106	38.4	(35.5–41.3)	19.1	(13.8–24.4)	<0.001	721	40.5	(36.9–44.1)	24.8	(18.5–31.2)	<0.001
situation	Unemployed	178	6.2	(2.6–9.7)	27.5	(15.0–40.0)		222	12.5	(8.1–16.8)	44.1	(34.3–54.0)	
	Other	1594	55.4	(53.0–57.8)	27.1	(24.9–29.3)		836	47.0	(43.6–50.4)	31.9	(28.7–35.1)	
Income level	Medium–high	1705	65.2	(62.9–67.5)	19.3	(17.4–21.2)	<0.001	886	55.9	(52.6–59.2)	22.3	(19.6–25.0)	<0.001
	Low	908	34.8	(31.7–37.9)	33.6	(30.5–36.7)		699	44.1	(40.4–47.8)	38.5	(34.9–42.1)	
Occupation	Nonmanual work	1020	61.2	(58.2–64.2)	22.2	(16.7–27.6)	0.072	452	41.9	(37.3–46.4)	25.7	(17.7–33.6)	0.009
	Manual work	646	38.8	(35.0–42.5)	18.4	(11.5–25.4)		628	58.1	(54.3–62.0)	33.1	(26.7–39.5)	
Marital status	Single	792	27.3	(24.2–30.4)	21.2	(15.0–27.4)	<0.001	422	23.7	(19.7–27.8)	28.9	(20.9–37.0)	0.023
	Married	1666	57.4	(55.1–59.8)	22.7	(18.5–27.0)		1051	59.1	(56.2–62.1)	29.8	(24.7–34.8)	
	Divorced/separated	130	4.5	(0.9–8.0)	32.3	(18.2–46.5)		106	6.0	(1.5–10.5)	27.4	(11.1–43.6)	
	Widower	312	10.8	(7.3–14.2)	33.0	(23.9–42.1)		198	11.1	(6.8–15.5)	39.9	(29.1–50.7)	
Living with a	Yes	1729	59.7	(57.4–62.1)	22.4	(18.3–26.6)	0.039	970	54.6	(51.5–57.7)	29.0	(23.7–34.3)	0.121
partner	No	1165	40.3	(37.4–43.1)	25.8	(20.9–30.8)		807	45.4	(42.0–48.8)	32.5	(26.8–38.1)	
Social support	Good support	2643	91.6	(90.5–92.6)	21.0	(17.6–24.4)	<0.001	1719	96.8	(96.0–97.6)	29.5	(25.5–33.5)	<0.001
	Bad support	243	8.4	(4.9–11.9)	54.7	(46.3–63.2)		57	3.2	(0.0–7.8)	63.2	(47.4–78.9)	

(*) *p*-values of the Chi-square test to check the significance of the differences among categories.

**Table 3:** Frequencies (*n*), means and confidence intervals at 95% (95%CI) for the quantitative variables studied, according to the risk of poor mental health (GHQ−, GHQ+), by sex and year of the survey.

MEN		2005				2010			
		*n*	Average	95%CI	*p* *	*n*	Average	95%CI	*p* *
Num. chronic diseases	GHQ−	2386	0.65	(0.60–0.69)	<0.001	1270	0.78	(0.71–0.86)	<0.001
	GHQ+	460	1.71	(1.52–1.90)		432	1.59	(1.37–1.80)	
EUROQoL quality of life	GHQ−	2386	0.93	(0.92–0.93)	<0.001	1270	0.92	(0.91–0.93)	<0.001
	GHQ+	460	0.73	(0.70–0.75)		432	0.78	(0.75–0.80)	
**WOMEN**									
Num. chronic diseases	GHQ−	2212	0.93	(0.87–0.99)	<0.001	1234	1.18	(1.07–1.28)	<0.001
	GHQ+	694	2.43	(2.25–2.60)		543	2.27	(2.03–2.52)	
EUROQoL quality of life	GHQ−	2212	0.89	(0.88–0.90)	<0.001	1234	0.87	(0.86–0.88)	<0.001
	GHQ+	694	0.68	(0.66–0.70)		543	0.70	(0.67–0.72)	

(*) *p*-values of student’s *t* test to compare the averages among categories of the variable.

**Table 4:** Odds ratios (OR) and 95% confidence intervals (95%CI) of association between the risk of poor mental health (GHQ+) and the variables studied *.

	2005						2010					
	Simple Analysis			Adjusted Analysis			Simple Analysis			Adjusted Analysis		
	OR	95%CI	*p* **	OR	95%CI	*p* **	OR	95%CI	*p* **	OR	95%CI	*p* **
Age												
16–44	1			1			1			1		
45–64	1.4	(1.2–1.6)	<0.001	0.8	(0.7–1.0)	0.082	1.1	(0.9–1.3)	0.337	0.8	(0.6–0.9)	0.010
65–84	1.8	(1.5–2.1)	<0.001	0.4	(0.3–0.6)	<0.001	1.1	(0.9–1.4)	0.297	0.4	(0.3–0.6)	<0.001
≥85	3.5	(2.3–5.2)	<0.001	0.5	(0.3–0.9)	0.026	2.6	(1.7–4.1)	<0.001	0.4	(0.2–0.7)	0.003
Sex												
Man	1			1			1			1		
Woman	1.6	(1.4–1.8)	<0.001	1.2	(1.0–1.4)	0.020	1.3	(1.1–1.5)	0.001	1.1	(0.9–1.3)	0.225
Country of birth												
Spain	1			1			1			1		
Abroad	0.9	(0.7–1.1)	0.441	1.0	(0.8–1.4)	0.743	1.6	(1.3–1.9)	<0.001	1.6	(1.2–2.0)	<0.001
Num. nonmental chronic diseases	1.55	(1.49–1.61)	<0.001	1.26	(1.19–1.32)	<0.001	1.24	(1.20–1.29)	<0.001	1.08	(1.0–1.1)	0.003
EuroQoL score	0.02	(0.01–0.03)	<0.001	0.04	(0.03–0.07)	<0.001	0.04	(0.03–0.06)	<0.001	0.05	(0.03–0.10)	<0.001
Disability												
No	1			1			1			1		
Yes	3.8	(3.2–4.4)	<0.001	1.2	(0.9–1.5)	0.121	2.9	(2.5–3.4)	<0.001	1.3	(1.1–1.7)	0.014
Social support												
Good	1			1			1			1		
Bad	5.1	(4.1–6.2)	<0.001	3.4	(2.7–4.4)	<0.001	4.2	(2.9–6.2)	<0.001	2.3	(1.5–3.5)	<0.001
Living with a partner												
Yes	1			1			1			1		
No	1.2	(1.0–1.3)	0.015	1.2	(1.0–1.4)	0.061	1.3	(1.1–1.5)	0.001	1.1	(0.9–1.3)	0.281
Employment Situation												
Employed	1			1			1			1		
Unemployed	2.6	(2.0–3.4)	<0.001	2.1	(1.6–2.8)	<0.001	2.4	(1.9–2.9)	<0.001	1.8	(1.5–2.3)	<0.001
Other	2.1	(1.8–2.4)	<0.001	1.3	(1.0–1.5)	0.017	1.5	(1.3–1.8)	<0.001	1.0	(0.8–1.2)	0.981
Income level												
Medium–high	1			1			1			1		
Low	2.1	(1.8–2.4)	<0.001	1.3	(1.1–1.5)	0.007	2.3	(1.9–2.7)	<0.001	1.6	(1.3–1.9)	<0.001

(*) Only significant variables are included in any year in the adjusted analysis. (**) *p*-values of significance for the estimated OR.

**Table 5:** Population distribution (% Pop), observed prevalence (P_o_) of poor mental health risk in 2005 and 2010, expected prevalence (P_e_) in 2010, standardizing according to the 2005 population structure of the variables included and difference between observed and expected prevalence.

	MEN						WOMEN					
	2005		2010				2005		2010			
	%Pop	P_o_	% Pop	P_o_	P_e_	P_o_–P_e_	%Pop	P_o_	% Pop	P_o_	P_e_	P_o_–P_e_
Total	100.0	16.2	100.0	25.4	21.4	4.0	100.0	24.0	100.0	30.6	28.5	2.1
Age												
16–44	55.8	13.5	54.0	25.4	21.8	3.6	50.9	19.7	48.8	28.1	25.4	2.7
45–64	27.7	16.8	29.2	25.2	18.8	6.4	28.0	26.7	29.5	31.1	29.1	2.0
65+	16.6	24.2	16.8	25.8	24.5	1.3	21.1	30.1	21.7	35.5	35.2	0.3
Country of birth												
Spain	89.9	16.4	85.7	24.2	20.9	3.3	89.6	23.9	87.6	29.1	27.5	1.6
Abroad	10.1	16.1	14.3	32.5	26.0	6.5	10.4	21.5	12.4	40.9	37.7	3.2
Income												
Medium–high	68.7	12.6	56.6	16.4	15.6	0.8	65.2	19.3	55.9	22.3	22.9	-0.6
low	31.3	23.5	43.4	33.5	30.0	3.5	34.8	33.6	44.1	38.5	35.6	2.9
Employment situation												
Employed	66.1	11.5	51.6	20.2	19.3	0.9	38.4	19.1	40.5	24.8	23.8	1.0
Unemployed	4.5	34.4	16.4	37.3	34.3	3.0	6.2	27.5	12.5	44.1	39.1	5.0
Other	29.3	23.8	32.0	27.6	24.2	3.4	55.4	27.1	47.0	31.9	30.6	1.3

## Abbreviations

VC	Valencian Community
ESCV	Health Survey of Valencian Community
